# Implementation process of the Surgical Safety Checklist: integrative review^*^

**DOI:** 10.1590/1518-8345.2921.3104

**Published:** 2019-01-31

**Authors:** Maria Fernanda do Prado Tostes, Cristina Maria Galvão

**Affiliations:** 1 Universidade Estadual do Paraná, Colegiado de Enfermagem, Paranavaí, PR, Brazil.; 2 Universidade de São Paulo, Escola de Enfermagem de Ribeirão Preto, PAHO/WHO Collaborating Centre for Nursing Research Development, Ribeirão Preto, SP, Brazil.

**Keywords:** Perioperative Nursing, Review, World Health Organization, Patient Safety, Checklist, Health Services, Enfermagem Perioperatória, Revisão, Organização Mundial da Saúde, Segurança do Paciente, Lista de Checagem, Serviços de Saúde, Enfermería Perioperatoria, Revisión, Organización Mundial de la Salud, Seguridad del Paciente, Lista de Verificación, Servicios de Salud

## Abstract

**Objective:**

to analyze the evidence available in the literature on the process of implementing the Surgical Safety Checklist, proposed by the World Health Organization, in the practice of health services.

**Method:**

integrative review, the search for primary studies was performed in three relevant databases in the health area, and the sample consisted of 27 studies, which were grouped into three categories.

**Results:**

the synthesis of the evidence indicated the different strategies that can be adopted in the implementation process (introduction and optimization) of the Surgical Safety Checklist, and the facilitators and barriers that determine the success in using this tool.

**Conclusion:**

in health services, implementing the checklist is a complex and challenging process that requires effective leadership, clear delegation of responsibilities from each professional, collaboration between team members, and institutional support. The synthesis of the generated knowledge can assist nurses in decision making, especially in identifying strategies for the effective implementation of the Surgical Safety Checklist, since nursing has the potential to be a protagonist in the planning and implementation of best practices for patient safety.

## RESUMO

**Objetivo:**

analisar as evidências disponíveis na literatura sobre o processo de implementação da Lista de Verificação de Segurança Cirúrgica, proposta pela World Health Organization, na prática dos serviços de saúde.

**Método:**

revisão integrativa. A busca dos estudos primários foi executada em três bases de dados relevantes na área da saúde, sendo a amostra composta por 27 pesquisas, as quais foram agrupadas em três categorias.

**Resultados:**

A síntese das evidências indicou as diferentes estratégias que podem ser adotadas no processo de implementação (introdução e otimização) da Lista de Verificação de Segurança Cirúrgica, e os facilitadores e barreiras determinantes para o sucesso no uso dessa ferramenta.

**Conclusão:**

nos serviços de saúde, a implementação do checklist é um processo complexo e desafiador que exige liderança efetiva, delegação clara das responsabilidades de cada profissional, colaboração entre os membros da equipe e suporte institucional. A síntese do conhecimento gerado pode auxiliar os enfermeiros na tomada de decisão, principalmente na identificação de estratégias para a implementação efetiva da Lista de Verificação de Segurança Cirúrgica, uma vez que a enfermagem tem potencial para ser protagonista no planejamento e na implementação das melhores práticas em prol da segurança do paciente.

## RESUMEN

**Objetivo:**

analizar las evidencias disponibles en la literatura sobre el proceso de implementación del Listado de Verificación de Seguridad Quirúrgica, propuesto por la Organización Mundial de la Salud, en la práctica de los servicios de salud.

**Método:**

revisión integrativa, la búsqueda de los estudios primarios fue ejecutada en tres bases de datos relevantes en el área de salud, siendo la amuestra compuesta por 27 investigaciones, las cuales fueron agrupadas en tres categorías.

**Resultados:**

la síntesis de las evidencias indicó las diferentes estrategias que pueden ser adoptadas en el proceso de implementación (introducción y optimización) del Listado de Verificación de Seguridad Quirúrgica, los facilitadores y barreras determinantes para el éxito en el uso de esa herramienta.

**Conclusión:**

en los servicios de salud, la implementación del listado de verificación es un proceso complejo y desafiador que exige liderazgo efectivo, delegación clara de las responsabilidades de cada profesional, colaboración entre los miembros del equipo y soporte institucional. La síntesis del conocimiento generado puede auxiliar a los enfermeros en la toma de decisión, sobre todo en la identificación de estrategias para la implementación efectiva del Listado de Verificación de Seguridad Quirúrgica, una vez que la enfermería tiene un potencial para ser protagonista en la planificación e implementación de las mejores prácticas en beneficio de la seguridad del paciente.

## Introduction

Starting at the year 2008, the World Health Organization (WHO) has recommended the implementation of the Surgical Safety Checklist (SSC) in a surgical room to prevent adverse events, strengthen safety practices and improve the quality of care provided to the surgical patient globally^1^ ^-^ ^3^.

The SSC is subdivided into three phases, each corresponding to a specific moment in the normal flow of the surgical anesthetic procedure, namely: period before anesthetic induction (sign in), period before the surgical incision (time out), and period immediately after surgery closure (sign out). Each phase contains specific items^3^.

In the world context, SSC was implemented in different health services and in clinical practice. Among the benefits obtained with the use of this tool are the increase to detect potential adverse events, reduction of surgical complications, improvement of communication and teamwork^4^ ^-^ ^5^. In contrast, the way the SSC implementation process occurs can lead to incomplete or inconsistent execution of the tool and low compliance rate by the surgical team^6^. Consequently, the benefits in their employment may vary according to the effectiveness of this process^7^.

SSC is considered a difficult implementation tool with application, reliability and execution problems. Health professionals understand that their use may increase the safety of the surgical patient, but there is no complete understanding of the need for behavior change and the incorporation of its use into daily practice^8^.

In a recent literature review, the authors stated that in low- and middle-income countries there is a lack of research on SSC compared to the large number of studies conducted in high-income countries that made it possible to construct a robust body of evidence in relation to use of the tool in practice. Although some of this knowledge can be applied and transferred to low- and middle-income countries, there are specific issues regarding the implementation and use of the checklist in the context of these countries, such as: the introduction of LSVC use in health services that did not incorporate and other relevant practices such as the surgical counting process, surgical site marking and administration of antibiotics, as well as limited resources and cultural differences^9^.

In low- and middle-income countries, SSC is known and often available, but its use is not yet universally promoted or implemented, indicating the need for targeted efforts in teaching about the tool^8^.

With the purpose of synthesizing evidence that can help nurses’ decision making in the effective implementation of this tool, promoting the adhesion of health professionals and making it feasible to incorporate in practice, the objective of the present integrative review was to analyze the available evidence in the literature on the process implementation of the Surgical Safety Checklist, proposed by the World Health Organization (WHO), in the practice of health services.

## Method

The method of knowledge synthesis adopted was the integrative review. Five steps were taken: the elaboration of the research question (identification of the problem), search in the literature for primary studies, critical appraisal of the primary studies, analysis of the data and presentation of the review^10^.

The guiding question was “What are the available evidence in the literature about the process of implementation of SSC proposed by WHO in the practice of health services?” In order to construct this question, the PICO strategy was employed, being P (population), patient or problem (surgical safety checklist proposed by WHO), I (intervention or area of interest) in the case of implementation process, and for element O (outcome) were adopted: facilitators and barriers of the process implementation of the surgical safety checklist. It is emphasized that the element C (comparison between intervention or group) was not used due to the type of review.

In order to search for the primary studies, we selected the PubMed, CINAHL (Cumulative Index to Nursing and Allied Health Literature) and LILACS (Latin American and Caribbean Literature in Health Sciences) databases. In each database, the controlled descriptors were delimited (Medical Subject Headings-MeSH, CINAHL Headings, and Descriptors in Health Sciences) and the keywords were defined.

The controlled descriptors and keywords used were: a) PubMed: Checklist, Checklist/Utilization, Patient Safety (MeSH); Checklists, Surgical safety checklist, World Health Organization, Implementation, Barriers, Facilitators and Benefits (keywords); b) CINAHL: Checklists, Checklists/utilization, Patient Safety, World Health Organization (CINAHL Headings); Checklist, Surgical safety checklist, Implementation, Barriers, Facilitators, Benefits and c) LILACS: Checklist, Checklist/Utilization, Patient Safety (Health Sciences Descriptors); Checklist, Implementation, Difficulties, Facilitators, Benefits (keywords).

For each database a search strategy was developed with the controlled descriptors and keywords already mentioned (different crossings). As an example, the search strategy employed in the PubMed database was: 1) Checklist OR Checklists OR Surgical safety checklist AND Implementation OR Checklist/utilization AND World Health Organization, 2) Checklist OR Checklists OR Surgical safety checklist AND Barriers, 3) Checklist OR Checklists OR Surgical safety checklist AND Facilitators and 4) Checklist OR Checklists OR Surgical safety checklist AND Patient Safety. In the selected databases, the search for primary studies occurred in January and February 2016.

The selection criteria for primary studies addressed the process of implementation of SSC proposed by WHO, in the practice of health services, namely: e.g. studies whose authors investigated the strategies used for the introduction or optimization of SSC in the intraoperative period; ii. studies that addressed the facilitators and barriers of the SSC implementation process, published in English, Portuguese and Spanish, from January 2010 to December 2015. The delimitation of this period is justified to ensure adequate quantification of primary studies, since the inclusion of high volume of research may make it unfeasible to conduct an integrative review or introduce biases in the next steps of the method.

In the evaluation of the primary studies, the nomenclature related to the type of study indicated by the authors was maintained. When the type of study was not clearly described by the researchers, the analysis was based on the concepts about the scientific methodology of nursing researchers^11^.

According to the clinical question of the study, scholars proposed hierarchies of evidence, which were adopted in the present review to classify the strength of the evidence. Thus, the clinical question of the primary study may be of Intervention/Treatment or Diagnosis/Diagnostic Test, the strength of evidence can be classified into seven levels, the strongest being (level I), the evidence of systematic review or meta-analysis of all relevant randomized controlled trials. When the clinical question of Prognosis/Prediction or Etiology, the strength of evidence can be classified into five levels, the strongest (level I) consists of the evidence of synthesis of cohort or case-control studies. With regard to the clinical question on meaning, the strength of evidence can be classified into five levels, the strongest (level I) being the evidence of the meta-synthesis of qualitative studies^12^.

The data extraction from the primary studies was performed with an standard instrument and submitted to face and content validation by the authors (Brazilian nurses). This step was carried out by two independent review authors.

The data analysis of the integrative review was elaborated in descriptive form. A summary table containing the following information was prepared for each primary study included: title of the study, author(s), journal, year of publication, objective(s), sample detail, type of study, main results and conclusions. The organization of the data in this way allowed the grouping of the primary studies into three categories, namely: “implementation process: strategies for introducing SSC in health services” (n=15); “Implementation process: strategies to optimize the use of SSC in health services” (n=9) and “facilitators and barriers to the implementation of SSC in health services” (n=3), allowing the comparison of differences and similarities among the researches. After conducting all the stages of RI, the synthesis of the knowledge about the subject investigated (SSC implementation process in the health services) provides the nurses’ decision-making about an important practice for patient safety and identify knowledge gaps for conducting future research in perioperative nursing.

## Results

**Figure 1:**
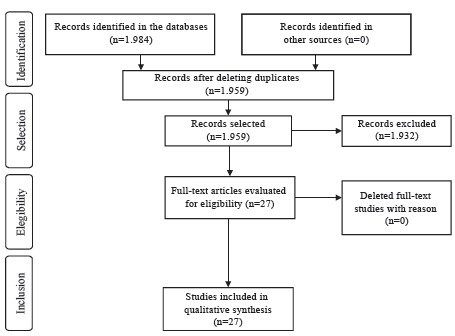
Flowchart of the selection process of primary studies adapted from the Preferred Reporting Items for Systematic Review and Meta-Analyses (PRISMA)

In the database search, we identified 1,984 potentially eligible studies (PubMed=1,124, CINAHL=808, LILACS=52). After reading the title and abstract of each publication, 25 were duplicated and were deleted. Of the total remaining (n=1,959), after applying the selection criteria, 1,932 were excluded, namely: eight studies in other languages, 102 were not primary studies and 1,822 did not address the SSC implementation process. Thus, the sample of the integrative review was composed of 27 primary studies, according to Figure 1. It should be emphasized that other sources of publications were not used, such as: manual search of the references for primary studies included in the review, as well as gray literature.

Of the 27 primary studies, 15 were classified according to the type of clinical question of Prognosis/Prediction or Etiology, being all with level of evidence IV; seven with type of clinical question of Intervention/Treatment or Diagnosis/Diagnostic Test, four being classified with level of evidence III, two level IV and one level VI. Of the five studies classified with clinical question of significance, four were level of evidence II and one level IV.

**Figure 2:**
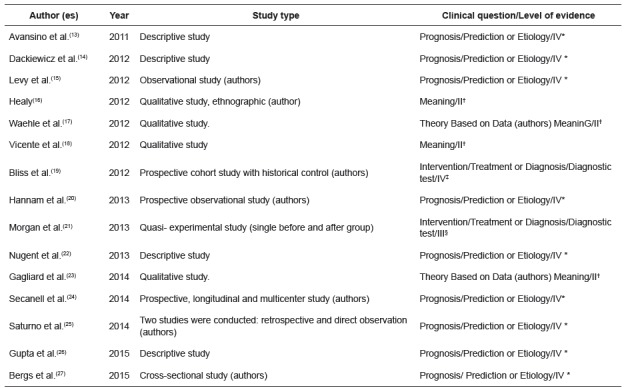
Characterization of primary studies, according to author (s), year of publication, type of study, clinical question and level of evidence, in the category implementation process: strategies for introduction of the Surgical Safety Checklist in health services n=15). Maringá, PR, Brazil, 2016

Figure 2 presents the characterization of the primary studies grouped in the first category “implementation process: strategies for the introduction of the Surgical Safety Checklist in health services” (n=15).

* IV-evidence of a single qualitative or descriptive study; ^†^ II - evidence of a single qualitative study; ^‡^ IV-evidence of well-delineated case-control and cohort studies; ^§^III - Evidence from well-delineated clinical trials without randomization

In the first category, the authors of the research investigated as main focus the strategies undertaken to introduce SSC in the operating room (n=15). The following are the strategies described in the primary studies, namely: composition of leadership team; planning; analysis of the local context; involvement of the target audience; adaptation of the SSC to the local context; dissemination; educational program; pilot test; audit; feedback/reminders and evaluation.

The composition of the leadership team consisted in identifying and inviting leaders to organize the team responsible for implementing SSC in the operating room^13^ ^-^ ^21^ ^,^ ^23^ ^-^ ^25^.

Among the studies listed, the researchers reported on how leadership was exercised, that is, local leadership committed to pedagogically influence the team with active presence in surgical rooms, encouraging peer adherence and ensuring the use of SSC^13^, and the inclusion of other leaders during the process^16^. In a primary study in the operating room environment, scholars described that nurses coordinating SSC screening exercised active leadership, control, and required staff attention in verbal check^17^.

In six primary studies, a team with different professional categories (managers from health departments, safety/quality professionals, surgeons, nurses, and other professionals) was composed to lead the SSC implementation process^14^ ^-^ ^16^ ^,^ ^20^ ^-^ ^21^ ^,^ ^25^. In three researches, nurses were the main leaders of this process^17^ ^-^ ^18^ ^,^ ^23^.

In only two primary studies, the researchers indicated the planning strategy for the introduction of SSC in the operating room. The authors described the strategic phase to plan the implementation of the tool^18^, and in the other study, scholars highlighted the existence of the planning stage, and those responsible for conducting this strategy informed that there was little time to plan and test the tool^23^.

The analysis of the local context, prior to the introduction of SSC, was adopted by the researchers of three primary studies, with different purposes, to obtain information about the occurrence of adverse events^13^; the safety practices adopted by the surgical team and the occurrence of surgical complications were analyzed^14^, and to diagnose the educational needs of the team^18^.

In six primary studies, involvement of the surgical team was mentioned: involvement of the team to adapt the SSC^14^ ^,^ ^17^; leaders committed to the team throughout implementation^16^; interviewing and composition of consensus groups for nurses to report difficulties in using the tool, and to propose changes^18^; engagement of the local team in the search for solutions to use the SSC^20^, and team meetings to share experiences^24^.

With the exception of one primary study^19^, in the other studies the researchers adopted the strategy of adapting the SSC in different ways: in the adaptation of SSC to the local context and to the specialty of pediatric surgery, the authors considered the occurrence of adverse events so that the tool could contemplate them^13^; simplification of SSC^16^ ^-^ ^18^; the checklist was integrated into the pre-existing break^20^; modification for outpatient surgery^19^; modified version to local reality^14^ ^-^ ^15^ ^,^ ^22^ ^-^ ^25^. In two primary studies, the authors investigated the strategy itself, similar results in both indicated that modifications made to the original WHO version varied among hospitals, and most health services excluded essential items from the checklist^26^ ^-^ ^27^.

In eight primary studies, the SSC dissemination/dissemination strategy was approached; the actions undertaken were: newsletters^13^ ^,^ ^24^; poster set in each operating room^13^ ^-^ ^15^; presentation of videos^13^ ^-^ ^15^; information sent by the hospital intranet, posters located in the area of anesthetic induction and instruction manual^14^; presentation of the SSC in a computer^15^; use of printed matter^20^; copies of the updated version of the tool available in operating rooms^21^; e-mail to surgical staff^23^ and use of posters^24^. SSC messages were disseminated by the leaders of the implementation process^13^, and continuous information was disseminated through conferences, phone calls and meetings^24^.

With regard to the educational program, the types of teaching strategies, materials used, frequency of achievement, content addressed and participating professional categories were different among the 12 primary studies^13^ ^-^ ^16^ ^,^ ^19^ ^-^ ^25^. The teaching strategies adopted in the educational programs consisted of training, workshops, e-learning, meetings, integration program for new hires and permanent education, interactive seminars, discussion forum and discussion in the operating room, meetings for joint learning experiences/ideas) between hospital representatives, clinical case presentation and conferences.

With regard to participants in educational programs, the authors mentioned the leaders of the SSC implementation process; all professional categories involved; multidisciplinary team; education by surgical specialty; with the exception of the medical category, the participation of the other categories was mandatory; participation of almost all of the nursing team and partial of the doctors^13^ ^-^ ^16^ ^,^ ^19^ ^-^ ^25^.

In the educational programs, the content covered information about external experiences with the use of SSC^14^ ^,^ ^18^; correct use of the checklist^15^ ^,^ ^18^ ^-^ ^20^ ^,^ ^24^ ^-^ ^25^; approach to SSC, without specifying topics^15^; protocol of tool use with emphasis on objectives^16^ ^,^ ^18^ ^-^ ^20^ ^,^ ^24^ ^-^ ^25^; results of the pilot test performed previously^14^; thematic communication and how to deal with barriers^19^; key questions and doubts about the use of the checklist^24^ and definition of roles, responsibilities and suggestions^25^.

In three primary studies, the researchers mentioned a pilot test performed in some pediatric subspecialties and, after six months, the SSC was fully implemented^13^; The pilot test was carried out for three months^14^, the nurses responsible for the implementation reported little time to test the checklist^23^.

In thirteen primary studies, the audit was a strategy adopted for the introduction of SSC in the operating room, which occurred by direct observation^14^ ^-^ ^21^ ^,^ ^23^ ^-^ ^25^; documentary analysis of records^13^ ^-^ ^14^ ^,^ ^16^ ^,^ ^19^ ^,^ ^20^ ^,^ ^23^ ^,^ ^25^; self-report through questionnaire completion^13^ ^,^ ^15^; interviews and focus group^17^; group interviews and consensus groups^18^, and collaborative meeting^24^. Only in a primary study, the method of data collection for audit was not mentioned^22^.

The use of feedback as a strategy occurred through the monthly disclosure of surgical team performance in SSC use^13^; presentation of the results obtained in the situational diagnostic phase of each specialty, and presentation of the results of the pilot test in workshops with analysis of errors and deficiencies in data recording^14^; to adapt the tool, data were provided by the surgical team to the leaders of the implementation process^17^; the benefits and difficulties perceived by the nurses were reported^18^; information on SSC use was given to surgical staff^20^; only a few hospitals that participated in the research used this strategy, and in these services there was little feedback (some reported/discussed individually)^23^. The use of the feedback facilitated the knowledge of the performance/adherence of the surgical team in real time^24^.

The use of reminders was performed in a different way, such as: poster installation in the operating room, promoting verbal interaction between leaders and their peers in loco^13^; posters in the anesthetic and computer room^14^; in each operating room, a poster was installed with information on the timing of the check and the required participants^15^; periodically, reminders to the team through a face-to-face conversation with surgeons and SSC applicants in order to remind them of the completion of the checklist^16^; posters were distributed to encourage the application of the tool and marking the surgical site according to the recommendations established in the protocol^24^.

In the evaluation of the implementation process for the introduction of SSC in health services, in 12 primary studies, the authors described the combination of different strategies (multifaceted approach) as a recommended way to ensure the use of the checklist, as well as the production of beneficial effects in clinical practice^13^ ^-^ ^18^ ^,^ ^20^ ^-^ ^25^. On the other hand, in three studies, the researchers described the use of specific strategies, namely: educational program^19^, and adaptation to the local context^26^ ^-^ ^27^.

**Figure 3:**
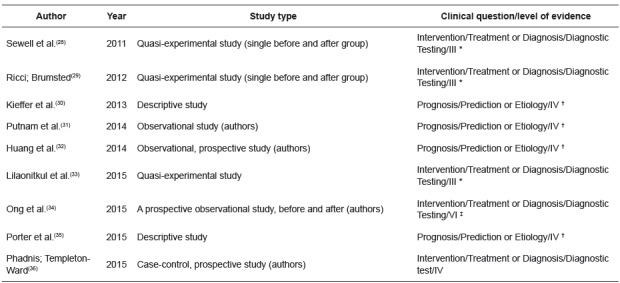
Characterization of primary studies, according to author (s), year of publication, type of study, clinical question and level of evidence, in the category of implementation process: strategies for optimization of the Surgical Safety Checklist in health services (n=9). Maringá, PR, Brazil, 2016

Figure 3 presents the characterization of the primary studies grouped in the second category “implementation process: strategies to optimize the use of the Surgical Safety Checklist in health services” (n = 9).

*III-evidence of well-delineated clinical trials without randomization; ^†^ IV-evidence of a single qualitative or descriptive study; ^‡^ VI-evidence of a single descriptive or qualitative study; ^§^IV-evidence of well-delineated case-control and cohort studies

In the second category, researchers from the primary studies investigated as a primary focus the strategies undertaken to improve SSC use in hospitals (n=9). To improve the practice of SSC use, the strategy of team composition/leadership recruitment occurred as follows: the authors mentioned the creation of a safety council coordinated by a physician and made up of members of the surgical and administrative team, which elaborated multifaceted and interdisciplinary strategy to be conducted by the leadership of the medical and nursing team^31^; quality improvement project was conducted by anesthesia resident under the leadership of senior consultant^33^; implementation of the tool by the change team and consultation with the leaders of each surgical discipline for engagement^34^; Quality improvement project was developed by multidisciplinary task force, and led by two surgeons and an anesthetist^35^.

The planning was made explicit in two primary studies, namely: strategic plan was adopted to develop multifaceted and interdisciplinary strategy to increase the use of SSC^31^; quality improvement methodology and Plan-Do-Study-Act cycles^33^.

Institutional support was indicated in two studies through involvement of the central administration to provide needed materials and equipment^33^; participation in the multidisciplinary meeting, in which the results on the performance of the team in the accomplishment of the preoperative instructions and association with intraoperative adverse events were disclosed, and provided support in the wide dissemination of the results obtained for all the personnel of the health institution^36^.

With the exception of one primary study^32^, in the others the researchers performed preliminary analysis of the local context before the implementation plan of strategies to optimize the application of the checklist. Preliminary analysis of the local context occurred through direct observation of clinical practice and/or analysis of data records, allowing the identification of problems: low adherence to use and/or inadequate performance in checking SSC items^(28-31,33 -36)^; lack of appropriation of the tool by the team^30^; lack of team engagement^34^, and occurrence of adverse events^28^ ^,^ ^33^ ^,^ ^36^. In five studies, the researchers emphasized that the way the checklist was introduced in the operating room contributed to the distortion of its use, for example, implementation of SSC in a taxing way^29^; limited implementation strategies^30^ ^,^ ^34^; lack of planning and other actions for introduction^31^ ^);^ leadership during the implementation process^33^.

In three primary studies, the researchers adopted the involvement of the target audience, as a strategy to improve the practice of using SSC, namely: consultation with the members of the surgical team to define the protocol for checking the tool^30^; the team assisted in the adaptation of the checklist^31^; the multidisciplinary discussion on facilitators and barriers to SSC use^33^.

The SSC adaptation was mentioned by the authors in three researches: adaptation of the design of the tool for pediatric surgery performed by the surgical team during a pedagogical workshop^31^; adaptation of the checklist with the standardization of instruments (on the back of the document) for the conference and registry of the surgical count^33^; revision of the content of the SSC by the multidisciplinary team of the surgical room^35^.

To optimize the use of SSC through dissemination, the implementation team developed a multimedia program and poster display^31^; posters were attached to the wall of the operating room in all specialties^33^ ^-^ ^35^, and drafting of instructional script^36^.

All authors of the primary studies included in this category used education as a strategy to improve SSC use. The educational program, as well as teaching strategies, educational materials, categories of participants, duration, frequency, contents were discussed and diverged among them^28^ ^-^ ^36^.

The pilot test was mentioned in three studies, in view of the inadequate use of SSC, the strategy was used in obstetric surgeries for reintroduction of the tool in a surgical room^33^; pilot test (two months) to test new format and definition of responsibilities in checking among professional categories^34^; revision and changes in the checking process, content of the checklist and definition of responsibilities were actions investigated in the pilot test (three months) in six operating rooms^35^.

The audit was performed in all the studies, by obtaining process indicators (adherence to the use of the tool), results (surgical complications and mortality) and the team’s perception about SSC^28^ ^-^ ^36^.

Regarding the feedback strategy and reminders, in the face of low membership by team members, individualized feedback was undertaken^31^; industry-specific charts with information on individual and team performance on the use of SSC^31^ and the installation of reminders (posters in operating rooms)^33^ ^,^ ^35^; Reminders on key changes were distributed to the team^34^; the information provided by the surgical team led to the adaptation of the tool. After the implementation of a quality improvement project, the team received feedback on the use of the checklist, published in a surgical forum^35^. Prior to the intervention (preoperative instructions), at a specialty meeting, feedback from the professionals’ performance was disclosed to the medical director, chief administrative officer and members of the surgical team^36^.

In the evaluation of the process of implementing strategies to optimize the use of SSC in health services, in five studies, the authors mentioned the use of a multifaceted approach^31^ ^,^ ^33^ ^-^ ^36^ and the use of a single strategy was adopted in four studies^28^ ^-^ ^30^ ^,^ ^32^.

**Figure 4:**
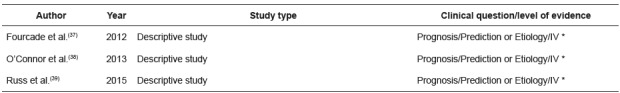
Characterization of primary studies, according to author, type of study, clinical question and level of evidence, in the category of facilitators and barriers for the implementation of the Surgical Safety Checklist in health services (n=3). Maringá, PR, Brazil, 2016

Figure 4 presents the characterization of the primary studies grouped in the third category “facilitators and barriers to the implementation of the Surgical Safety Checklist in health services” (n = 3).

*IV- evidence from a single qualitative or descriptive study

In the third category, the authors of the primary studies investigated as the main focus the facilitators and barriers of the SSC implementation process in health services (n=3).

In one primary study, the facilitators listed were the conviction of some experienced physicians about the relevance of SSC, promoting their use more effectively; leadership of experienced surgeons and anesthetists conducting tool check; involvement of the multidisciplinary team; management support; simplification of checklist items; involvement of the team in the implementation of the tool; education and training, feedback, sanctions applied in the absence of adhesion and adaptation of the checklist to better integrate the work process^39^.

In the primary studies, the barriers presented were: lack of understanding about the items and adequate moment for checking^37^; lack of understanding about the benefits of checklist^37^ ^-^ ^39^; poor communication between the categories of surgeons and anesthetists, hierarchy among professional categories^37^ ^-^ ^38^; absence of teamwork and senior support^38^; active or passive resistance of some professionals, especially those more experienced, with greater frequency of surgeons and anesthetists, and skepticism regarding the evidence base on SSC^39^.

In addition, the time spent with the check consisted of a barrier^37^ ^,^ ^39^; ambiguous list check items and unaccounted risks, ie the checklist did not contain items that included other care that should be performed to prevent adverse events or complications in the patient (eg, preparation of the patient’s skin)^37^; in addition to the routine of filling out different forms and signatures, the need for another form to register checklist data, lack of time for checking and carrying out simultaneous activities during its execution, absence of educational process/orientation, and need for signatures of the team members in completing SSC^38^; institutional culture resistant to change, procedures to be performed in SSC redundant checking with existing practices, creating difficulties for the integration of the tool into the work process and implementation without planning or imposition, very long checklist, content and layout of the tool, inappropriate items for certain procedures, specialties and contexts, SSC items that require verbal confirmation by the patient give a false impression that the surgical environment is unsafe, generating anxiety^39^.

## Discussion

The evaluation of the strategies used in the SSC implementation process (introduction and optimization) was analyzed in all the primary studies grouped in the first and second categories. In the first category, in seven studies^13^ ^-^ ^14^ ^,^ ^16^ ^-^ ^18^ ^,^ ^22^ ^,^ ^24^ the authors emphasized that the implementation of the tool was considered successful and recommended, and had beneficial effects for the clinical practice, surgical team and patient, education being the key element in this process.

On the other hand, in two primary studies, the results showed that the strategies used were successful in some aspects, and failed in others, for example, the realization of a structured educational program (low cost intervention) allowed a significant reduction in morbidity and costs, but there was persistence in the variation of adherence to SSC use and communication failures(19). The strategies defined by the WHO to implement the tool in a pilot hospital contributed to the improvement of the adherence of the professionals, but there was no increase in adherence at all stages (before anesthetic induction, before the surgical incision and immediately after the surgical incision closure)^20^.

In four primary studies, the adopted strategies did not produce the expected effects, resulting in a lack of fidelity in the daily use of SSC^15^; although the checklist was adapted for outpatient surgery, its use did not contribute to the reduction of postoperative complications^21^, and the mandatory use of the tool did not promote the improvement of the safety culture^23^ ^,^ ^25^. In two studies, the authors suggested that the SSC’s local adaptation strategy, excluding items from the original version proposed by the WHO, may hamper the achievement of benefits for the surgical patient^26^ ^-^ ^27^.

In the second category, in seven primary studies^29^ ^-^ ^35^, the adopted strategies were considered successful, promoting the reduction of adverse events (for example, surgery in the wrong place and retention of surgical items), improved adherence to the use of the tool and the execution of the surgical counting process, an increase in safety culture and the strengthening of teamwork.

In two primary studies, the implementation of strategies to optimize SSC use in two primary studies has had beneficial effects, but has not achieved other desired results, ie, despite improved adherence to tool use and team perception of the checklist, there was a significant improvement in the results for the patients^28^. In another study, the adopted intervention (pre-operative instructions) improved the quality of execution of SSC use by the surgical team, and a statistically significant reduction of adverse events, however, the complete preoperative instructions were not performed in all observed cases^36^.

In the first two categories delimited, based on the results of the research, it can be inferred that, in the majority, the authors investigated the adoption of combined (multifaceted) strategies for the implementation process or optimization of SSC use, which were successful, producing beneficial or expected results in clinical practice, surgical team and patient.

With regard to the third delimited category, the knowledge about facilitators and barriers of the SSC implementation process can contribute to support the planning of more adequate strategies and plays an important role in determining the success of the implementation of this tool in the health services.

In the conduct of this integrative review, the evidence generated provides insights for the understanding of the SSC implementation process, different strategies that can be used, and the aspects of implementation considered successful or not very successful in achieving the expected results. Thus, the knowledge produced can contribute to the improvement of the safety culture of the patient, a reality necessary in the national context^40^.

Regarding the limitations of the present review, the authors delimited published primary studies, that is, the gray literature was not included, as well as language restriction. The data analysis was performed in a descriptive way, so the combination of data from different types of studies (quantitative and qualitative methodological approach)could be done, thus it is a complex process that can lead to bias in the elaboration of the results of the review.

## Conclusion

In the health services, the implementation of SSC is a complex and challenging process that requires the involvement of all the health professionals responsible for the care of the patient in the intraoperative period. For the successful implementation of this tool there is a need for effective leadership, clear delegation of responsibilities of each professional, collaboration between the team members and institutional support providing human resources and materials necessary for the daily use of the checklist.

In most of the primary studies included in the review, there was not enough description of the strategies employed, which made it impossible to know the actions developed in each strategy. Thus, in conducting future research it is recommended that the actions carried out be described in detail to assist health professionals in understanding the SSC implementation process.

Nurses can use the results of this review for decision-making in the selection and implementation of appropriate strategies for effective implementation of SSC, since nursing has the potential to be a key player in planning and implementing best practices for patient safety.
